# Temporal expression dynamics of lncRNAs and cis-target gene interactions in *Leishmania major*-infected human macrophages

**DOI:** 10.1097/MD.0000000000044129

**Published:** 2025-08-29

**Authors:** Serhat Sirekbasan, Tuğba Gürkök-Tan

**Affiliations:** a Department of Medical Services and Techniques, Şabanözü Vocational School, Çankiri Karatekin University, Çankiri, Turkey; b Department of Field Crops, Food and Agriculture Vocational School, Çankiri Karatekin University, Çankiri, Turkey.

**Keywords:** cis-target genes, gene expression, *Leishmania major*, long non-coding RNAs (lncRNAs), macrophages

## Abstract

Leishmaniasis is a serious infectious disease caused by *Leishmania* parasites, predominantly affecting tropical and subtropical regions. These parasites replicate within macrophages, manipulating the host immune response and facilitating infection progression. While long non-coding RNAs (lncRNAs) are known regulators of immune function, their time-dependent roles during *Leishmania major* infection remain unclear. Specifically, we now highlight that this is the first time the temporal dynamics of lncRNA expression and cis-target gene interactions have been systematically analyzed in *L major*-infected macrophages across 4 distinct time points. This study examined the expression profiles of long non-coding RNAs (lncRNAs), potential cis-target genes, and hub genes at different time points in human macrophages infected with *L major*. RNA-Seq analysis identified 39,828 lncRNAs, with 2903 showing differential expression at one or more time points. As the infection progressed (4, 24, 48, and 72 hours), the number of up- and down-regulated lncRNAs showed a dramatic decrease between 24 and 48 hours, followed by a slight increase between 48 and 72 hours. Six lncRNAs (*lnc-UNC5D-8*, *lnc-TENM3-1*, *DIRC3-1*, *lnc-MTRNR2L12-10*, *lnc-FAM43A-6*, and *AKAP2-1*) were consistently differentially expressed throughout the infection timeline and may play critical roles in modulating the host immune response. Time-specific hub genes were also identified, regulating critical processes such as keratinization, epigenetic modifications, and immune responses. In particular, these genes were pivotal during the later stages of infection in maintaining tissue integrity and regulating immune responses. Early immune responses were dominated by immunoglobulin receptor activity and adaptive immune system activation. These findings highlight the critical roles of lncRNAs and hub genes in macrophage responses to *Leishmania* infection, offering potential targets for future therapeutic strategies.

## 1. Introduction

Leishmaniasis is an infectious disease caused by *Leishmania* parasites that predominantly affects tropical and subtropical regions and has significant effects on human health.^[1]^ These parasites multiply in host macrophage cells and cause the disease to progress. Macrophages are an important component of the host immune system and regulate various immune responses when they encounter pathogens to fight infection.^[2]^ However, *Leishmania* parasites are capable of manipulating the defense mechanisms of macrophages, facilitating their own replication and contributing to the advancement of the infection.

During *Leishmania* infection, macrophages undergo significant changes in various biological processes. Both host and parasite experience alterations in gene expression throughout the course of the infection.^[3]^ Identifying the epigenetic changes induced by the parasite over time in the host can provide valuable insights into the progression of the disease. In particular, elucidating the mechanisms involved in regulating gene expression within macrophages can enhance our understanding of the pathogenesis of the infection and contribute to the development of novel therapeutic strategies.^[4]^

In recent years, non-coding RNAs (ncRNAs) have emerged as promising tools in both the diagnosis and treatment of infectious and noninfectious diseases, making them a focal point of diagnostic research.^[5]^ In eukaryotic cells, ncRNAs can be classified into 3 groups based on their nucleotide sequence length, function, and structure: small RNAs (<50 nucleotides), RNA polymerase III transcripts (e.g., 5S rRNA, tRNA), and long non-coding RNAs (lncRNAs; >200 nucleotides).^[6]^ LncRNAs are distinguished from other non-coding RNAs by their characteristics, including small open reading frames, low GC content, tissue-specific expression, and poorly conserved sequences across species.^[7]^ Based on their genomic location, lncRNAs can be categorized into 4 groups: sense, antisense, intronic, and intergenic.^[8]^

In mammals, thousands of lncRNAs have been shown to play regulatory roles in various biological functions, such as cell development,^[9]^ chromatin modification,^[10]^ and immune regulation.^[11]^ LncRNAs can interact with RNA, DNA, proteins, or microRNAs (miRNAs) to regulate processes including transcription, splicing mechanisms, nucleic acid degradation, and translation. Additionally, lncRNAs are involved in regulating gene expression in developmental processes, cell proliferation, inflammation, and host-pathogen interactions.^[12]^

Although several transcriptomic studies have examined the relationship between mRNAs and lncRNAs in macrophages infected by *Leishmania* species,^[13]^ these studies have not identified the target genes of lncRNAs involved during a specific time course of *L major* infection. Therefore, this study aims to identify the time-dependent expression patterns of lncRNAs and their potential cis-regulatory effects in human macrophages infected with *L major*. We hypothesize that specific lncRNAs reveal dynamic temporal expression profiles and regulate immune-related pathways during different stages of infection.

## 2. Materials and methods

### 2.1. RNA-seq data retrieval from macrophages infected with Leishmania major

To investigate and compare the lncRNA expressions in *L major* (Friedlin strain)-infected human macrophages, previously generated public RNA-Seq data were downloaded from the SRA–NCBI database under BioProject number PRJNA290995. The PRJNA290995 dataset was selected due to its high sequencing depth, biological replication (n = 4 per group), and consistent experimental design. Data quality and reproducibility were ensured by applying FastQC and selecting datasets with uniform read lengths and sequencing platforms. The libraries were sequenced by Fernandes et al using the Illumina HiSeq 1500 platform.^[14]^ PRJNA290995 contains a total of 66 transcriptome libraries. Here we selected the data sets belonging to *L major*. The datasets we selected consisted of 4 different time periods (4 hours, 24 hours, 48 hours, and 72 hours) after infection of macrophages and also uninfected groups corresponding to each treatment (Table S1, Supplemental Digital Content, https://links.lww.com/MD/P769).

### 2.2. Bioinformatic identification of lncRNAs and gene expression analysis

RNA-Seq files for both control and treated groups were downloaded from the Sequence Read Archive (SRA) database at NCBI (https://www.ncbi.nlm.nih.gov/). The raw reads adapters and low-quality reads were filtered out by fastqc v 0.11.9.^[15]^ After, the transcripts were mapped to human genome assembly GRCh38.p13 provided by GENCODE using the HISAT2 software v2.2.1.^[16]^ Whether the determined lncRNAs consisted of annotated lncRNAs or not, the structures of lncRNAs and mRNAs were compared according to transcript length, number of exons and ORFs. The coding potential of the filtered transcripts were predicted using lncRNA prediction tool CPC. The crossover data were coupled with known lncRNAs for further research. The abundances of transcripts were calculated as transcripts per million units.^[17]^ Differentially expressed lncRNAs (DElncRNAs) across different time points were identified using edgeR. Prior to modeling, TMM (Trimmed Mean of M-values) normalization was applied to adjust for differences in library size and sequencing depth. During the *P* value calculation, the model was created with the generalized linear model in the edgeR package.^[18]^ While creating this model, first a matrix was created on the basis of transcripts and groups, this matrix was also normalized and the model that calculates its distribution in groups was calculated. Then the fold change between time periods was compared and *P* value calculation was made for each transcript.^[19]^ Differentially expressed lncRNAs (DElncRNAs) were identified by applying both log2 fold change (log2FC) ≥ 1.5 or ≤ −1.5 and an adjusted *P*-value < .05.^[20,21]^ To correct for multiple testing, we used the Benjamini–Hochberg procedure to control the false discovery rate (FDR). To assess the robustness of the differential expression results, we conducted sensitivity analyses by varying the log2 fold change thresholds (e.g., ≥1.0 and ≥2.0) while keeping the adjusted *P*-value threshold constant (FDR < 0.05). These tests demonstrated that a substantial proportion of the DElncRNAs remained consistent across different cutoff criteria. Additionally, the differential expression analysis was repeated using quasi-likelihood F-tests within edgeR. The overlap of DElncRNAs across methods further confirmed the reliability of our results.

### 2.3. The cis-target gene identification and functional enrichment analysis

Given that the 300 kb threshold is a widely accepted criterion in lncRNA research^[22]^ a genomic region extending 300 kb upstream and downstream of each differentially expressed lncRNA was examined to identify potential cis-regulated mRNAs. Using the g:Profiler (https://biit.cs.ut.ee/gprofiler/gost, accessed on August 7, 2024), differentially expressed lncRNAs cis-target genes were submitted to gene ontology (GO) and pathway analysis to assess their roles in biological processes (BP), molecular function (MF), and cellular component (CC) terms. The parameter was filtered at an adjusted *P*-value of <.05.

### 2.4. PPI network construction and hub gene identification

PPI network was constructed for all time point comparisons from the cis-target genes odd DELs using STRING search tool.^[23]^ Then, Cytoscape (version 3.10.2) software was used for network visualization.^[24]^ Hub genes were identified using the CytoHubba plugin in Cytoscape, applying the Maximal Clique Centrality algorithm. Based on degree centrality, the top 10 key hub genes were identified.

## 3. Results

We re-analyzed RNA-Seq data generated by Fernandes et al to investigate the differentially expressed long non-coding RNAs (DElncRNAs) in human macrophages at 4-, 24-, 48-, and 72-hours post-infection (hpi) with *L major*. After processing control and time-point datasets, clean reads were aligned to the GRCh38.p13 genome assembly.

### 3.1. Differentially expressed lncRNAs between time point comparisons in human macrophages

**Figure 1.**
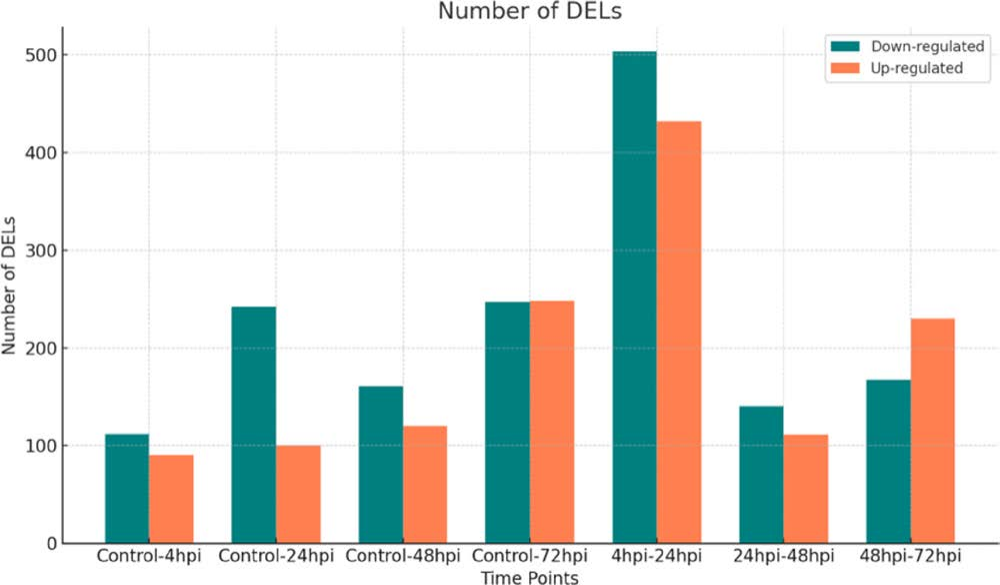
Temporal dynamics of DElncRNAs in *Leishmania*-infected macrophages across 4-, 24-, 48-, and 72-hpi compared to uninfected macrophages. DElncRNAs = differentially expressed long non-coding RNAs, hpi = hours post-infection.

In total, we detected 39.828 lncRNAs and classified them based on genomic location, with 77.54% identified as intergenic as shown in Figure 1 and detailed in Table S1 (Supplemental Digital Content, https://links.lww.com/MD/P769). Among these, 2.903 DElncRNAs were expressed in at least 1 of 7 comparisons (4 hpi vs control; 24 hpi vs control; 48 hpi vs control; 72 hpi vs control; 4 hpi vs 24 hpi; 24 hpi vs 48 hpi; 48 hpi vs 72 hpi). Specifically, 202, 342, 281, and 495 DElncRNAs were detected at 4-, 24-, 48-, and 72-hpi, respectively, compared to the uninfected samples (Fig. 1, Table S2, Supplemental Digital Content, https://links.lww.com/MD/P770). Additionally, 935, 251, and 397 DElncRNAs were identified in the time-point comparisons of 4 hpi vs 24 hpi, 24 hpi vs 48 hpi, and 48 hpi vs 72 hpi, respectively.

**Figure 2.**
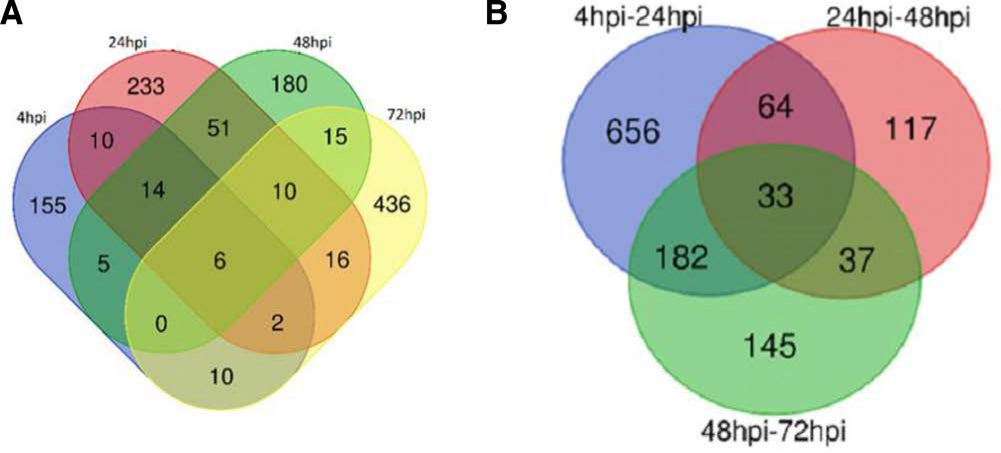
Venn diagrams of differentially expressed lncRNAs in *Leishmania*-infected macrophages. (A) Venn diagram illustrating the overlap of differentially expressed lncRNAs at 4 hpi, 24 hpi, 48 hpi, and 72 hpi time points in *Leishmania*-infected macrophages; (B) Venn diagram comparing the differentially expressed lncRNAs between the time intervals of 4 hpi to 24 hpi, 24 hpi to 48 hpi, and 48 hpi to 72 hpi in *Leishmania*-infected macrophages. hpi = hours post-infection.

There was no consistent pattern in the number of up- or down-regulated DElncRNAs over time when comparing each time point with its control. However, within time-point comparisons, the number of DElncRNAs showed a dramatic decrease from 24 to 48 hpi, followed by a small increase between 48 and 72 hours. Overlaps in DElncRNAs between comparisons are shown in Figure 2A and B. Notably, 6 lncRNAs (*lnc-UNC5D-8*, *lnc-TENM3-1*, *DIRC3-1*, *lnc-MTRNR2L12-10*, *lnc-FAM43A-6*, and *AKAP2-1*) were differentially expressed at all time points, with all but AKAP2-1 being consistently down-regulated.

Additionally, lnc-CMPK2-2 and lnc-TMEM121-26 showed expression changes across all time points. Table 1 illustrates the fold changes for both lncRNAs at different time intervals.

**Table 1:** Fold change in expression of lnc-CMPK2-2 and lnc-TMEM121-26 across different time intervals during *L major* infection.

Time interval	lnc-CMPK2-2	lnc-TMEM121-26
0 hpi–4 hpi	2.71	−2.48
4 hpi–24 hpi	3.61	5.38
24 hpi–48 hpi	2.36	−3.51
48 hpi–72 hpi	−4.10	−3.19

### 3.2. Cis-target genes of DElncRNAs and functional enrichment analysis

Cis-target genes of the identified DElncRNAs were located within 300 kb up-stream or downstream of each lncRNA’s transcription start and stop sites. A total of 58,446 cis-acting target genes were identified for the 2.903 DElncRNAs (Table S3, Supplemental Digital Content, https://links.lww.com/MD/P771).

Most lncRNAs’ functions have not been fully examined till now and analyzing cis-target genes of them using GO functional analysis and pathway analysis may help to identify lncRNA roles in the response upon *L major* infection in human macrophages. Cis-target genes were examined for KEGG pathway and GO enrichment in MF, BP and CC. Our aim in this study was to provide clues regarding the epigenetic regulation of human macrophages following *L major* infection across time points. As expected, we identified similarities as well as differences in the molecular response of the cell to the pathogen over time points. GO terms significantly enriched for cis-target genes of DElncRNAs at each time-point comparison are presented in Table S4 (Supplemental Digital Content, https://links.lww.com/MD/P772).

### 3.3. Temporal dynamics of lncRNA expression and immune response

In our study, we examined the expression profiles of lncRNAs in human macrophages infected with *L major* at different time points of infection (4, 24, 48 and 72 hours) and identified potential cis-target genes of these lncRNAs. Comparisons at different time points revealed significant changes in BP, CC and MF. These changes helped us understand how lncRNAs play a dynamic role in infected macrophages over time.

**Figure 3.**
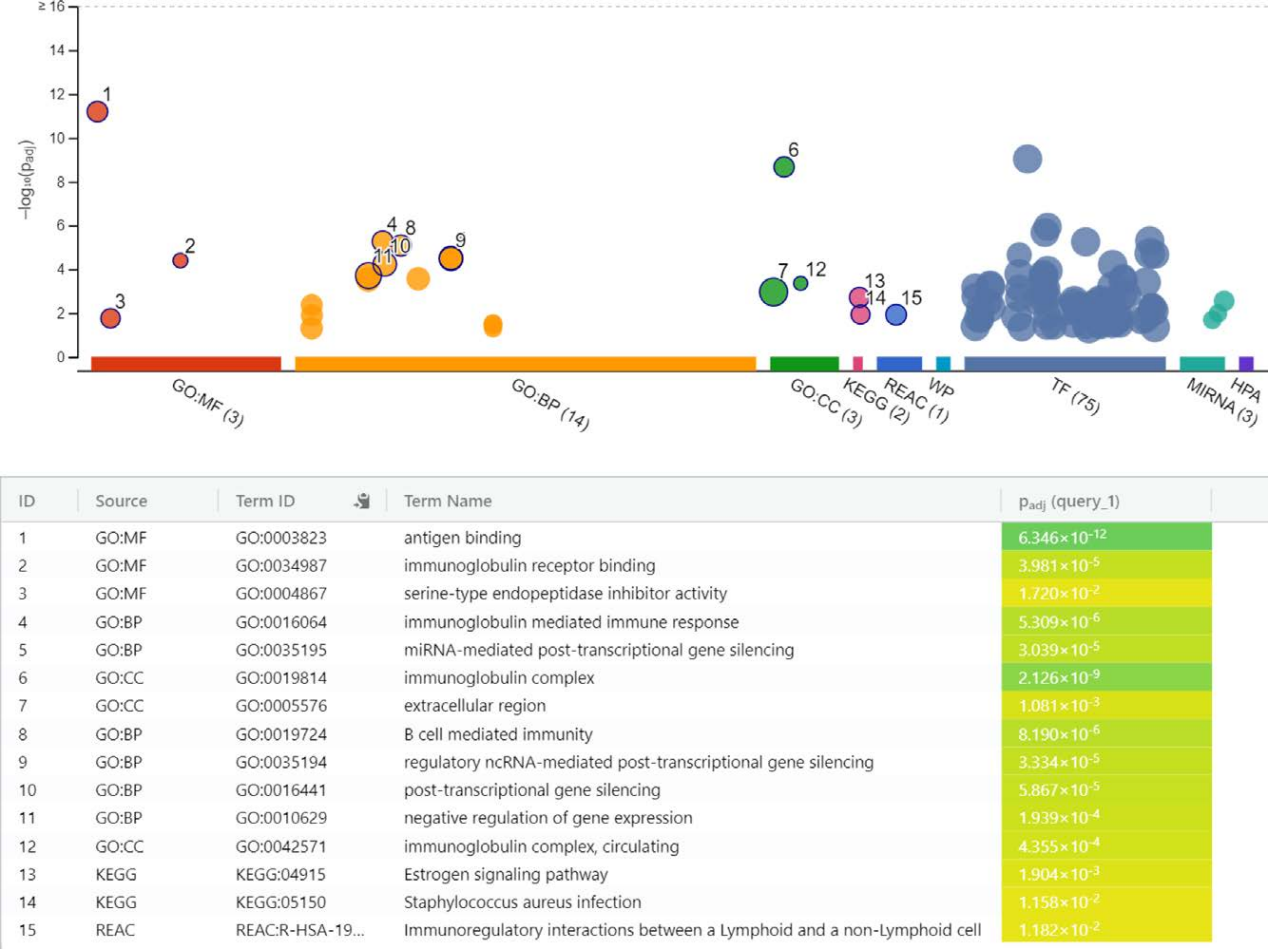
Functional enrichment of immune-related pathways and molecular components in *Leishmania*-infected macrophages at 0 to 4 hpi. BP = biological processes, CC = cellular components, GO = gene ontology, KEGG = Kyoto encyclopedia of genes and genomes, MF = molecular functions, REAC = reactome pathway database.

Firstly, a rapid activation of the immune response was observed in the early stages of infection compared to the control group at 4 hours of infection. During this period, processes associated with the activation of the adaptive immune system, such as the biological process immunoglobulin-mediated immune response and B cell-mediated immunity, were found to be prominent. In terms of CC, the plasma membrane and immunoglobulin complexes were active. In terms of MF, immune response triggering functions such as antigen binding and immunoglobulin receptor binding were found to be important. Enrichment analysis also highlighted pathways such as Estrogen signaling (KEGG:04915) and Immunoregulatory interactions (REAC:R-HSA-198933). This early response, as shown in Figure 3, may be regulated by 74 transcription factors and 3 microRNAs (hsa-miR-9500, hsa-miR-6813-5p, hsa-miR-6085) (Table S4, Supplemental Digital Content, https://links.lww.com/MD/P772).

**Figure 4.**
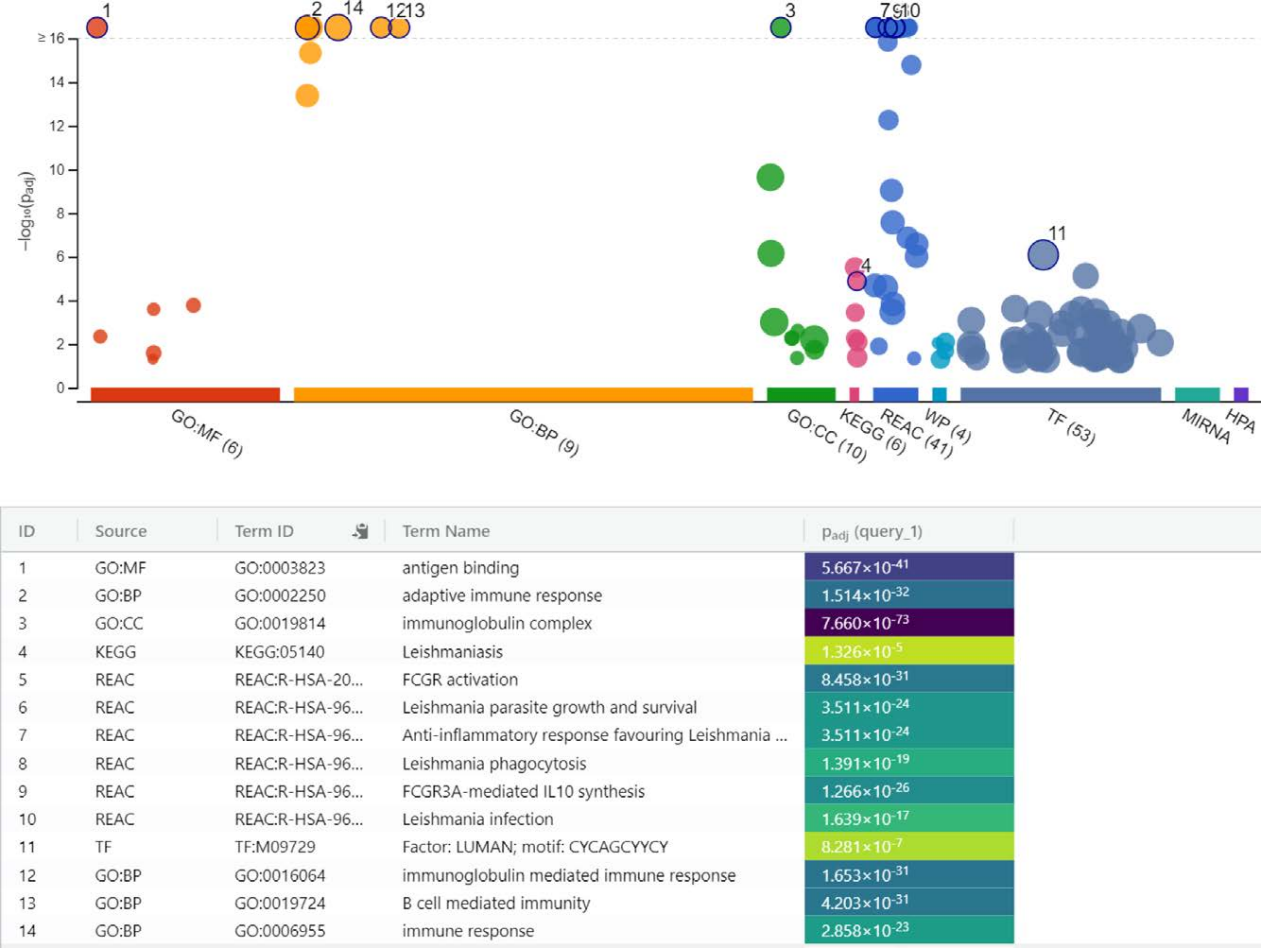
Enrichment analysis of immune pathways in *Leishmania*-infected macrophages between 4- and 24-hpi. BP = biological processes, CC = cellular components, GO = gene ontology, KEGG = Kyoto encyclopedia of genes and genomes, MF = molecular functions, REAC = reactome pathway database.

Between 4- and 24-hpi, lncRNAs played increasingly complex roles in immune regulation. MF related to chemokine receptors and proinflammatory cytokine release were regulated, while BP involving inflammatory responses became prominent. Several immune-related pathways, including those associated with Leishmaniasis (KEGG:05140, REAC:R-HSA-9664433, REAC:R-HSA-9662851, REAC:R-HSA-9664417, REAC:R-HSA-9658195) and immune signaling pathways, became statistically significant for the first time during this interval (Fig. 4, Table S4, Supplemental Digital Content, https://links.lww.com/MD/P772).

**Figure 5.**
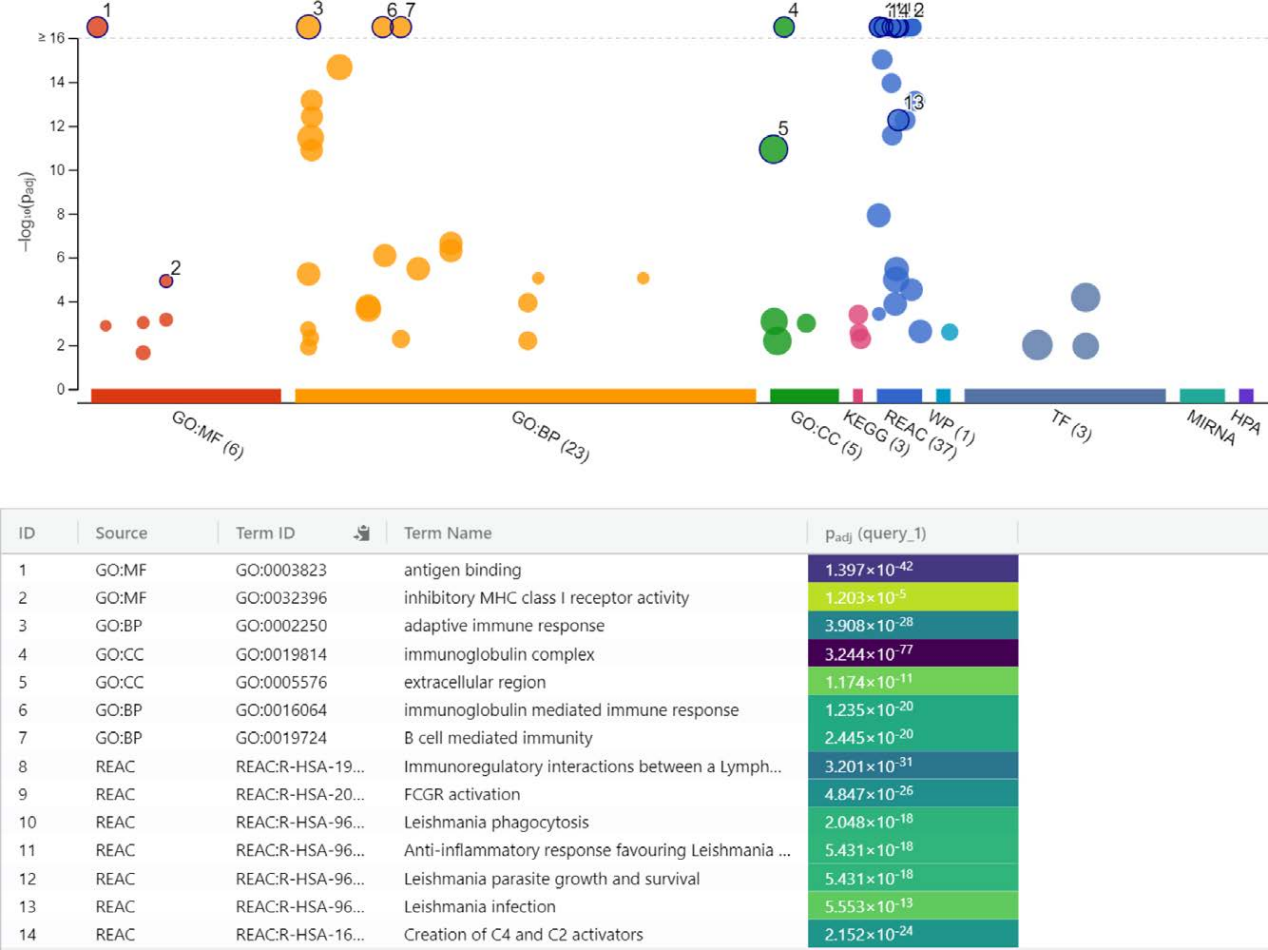
Identification of adaptive immune pathways and cellular components in macrophages between 24 and 48 hpi during *Leishmania* infection. BP = biological processes, CC = cellular components, GO = gene ontology, KEGG = Kyoto encyclopedia of genes and genomes, MF = molecular functions, REAC = reactome pathway database.

From 24 to 48 hours, significant changes in immune response were observed. BP such as adaptive immune response and post-transcriptional gene regulation were regulated, and CC including the immunoglobulin complex and keratin filaments were identified. Pathways related to B cell receptor signaling (KEGG:04662) and *Leishmania*-related phagocytosis were significantly enriched, as illustrated in Figure 5 (Table S4, Supplemental Digital Content, https://links.lww.com/MD/P772).

**Figure 6.**
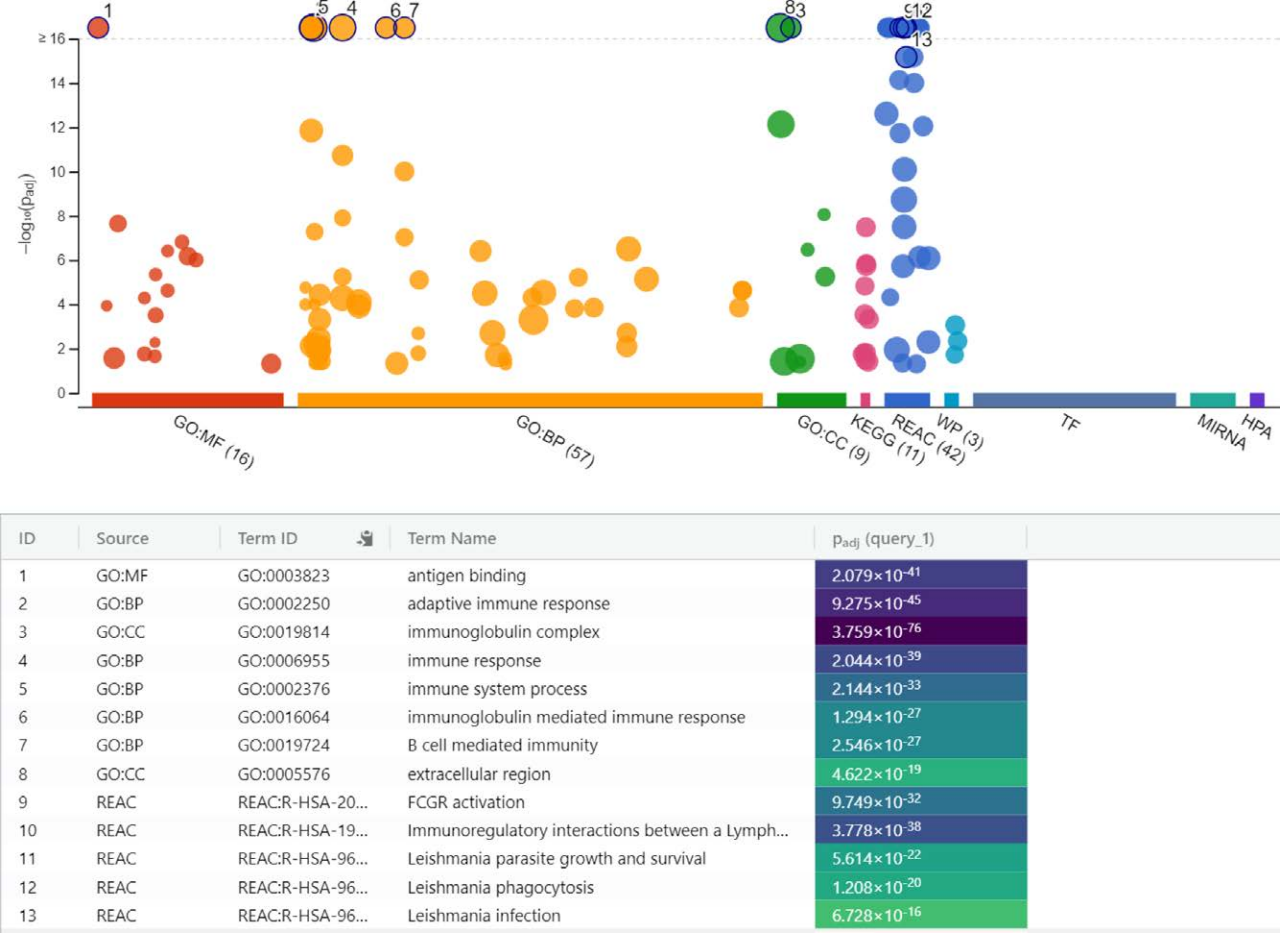
Enrichment of tissue remodeling, humoral immune responses, and chemokine activities in macrophages between 48 and 72 hpi during *Leishmania* infection. BP = biological processes, CC = cellular components, GO = gene ontology, KEGG = Kyoto encyclopedia of genes and genomes, MF = molecular functions, REAC = reactome pathway database.

Finally, between 48 and 72 hours, a more complex immune response emerged, with BP related to tissue remodeling, cell differentiation, and inflammation resolution being prominent. CC such as the extracellular matrix and cell surface receptors were active, and MF including chemokine receptor activity and signal transduction were highlighted (Fig. 6, Table S4, Supplemental Digital Content, https://links.lww.com/MD/P772).

**Figure 7.**
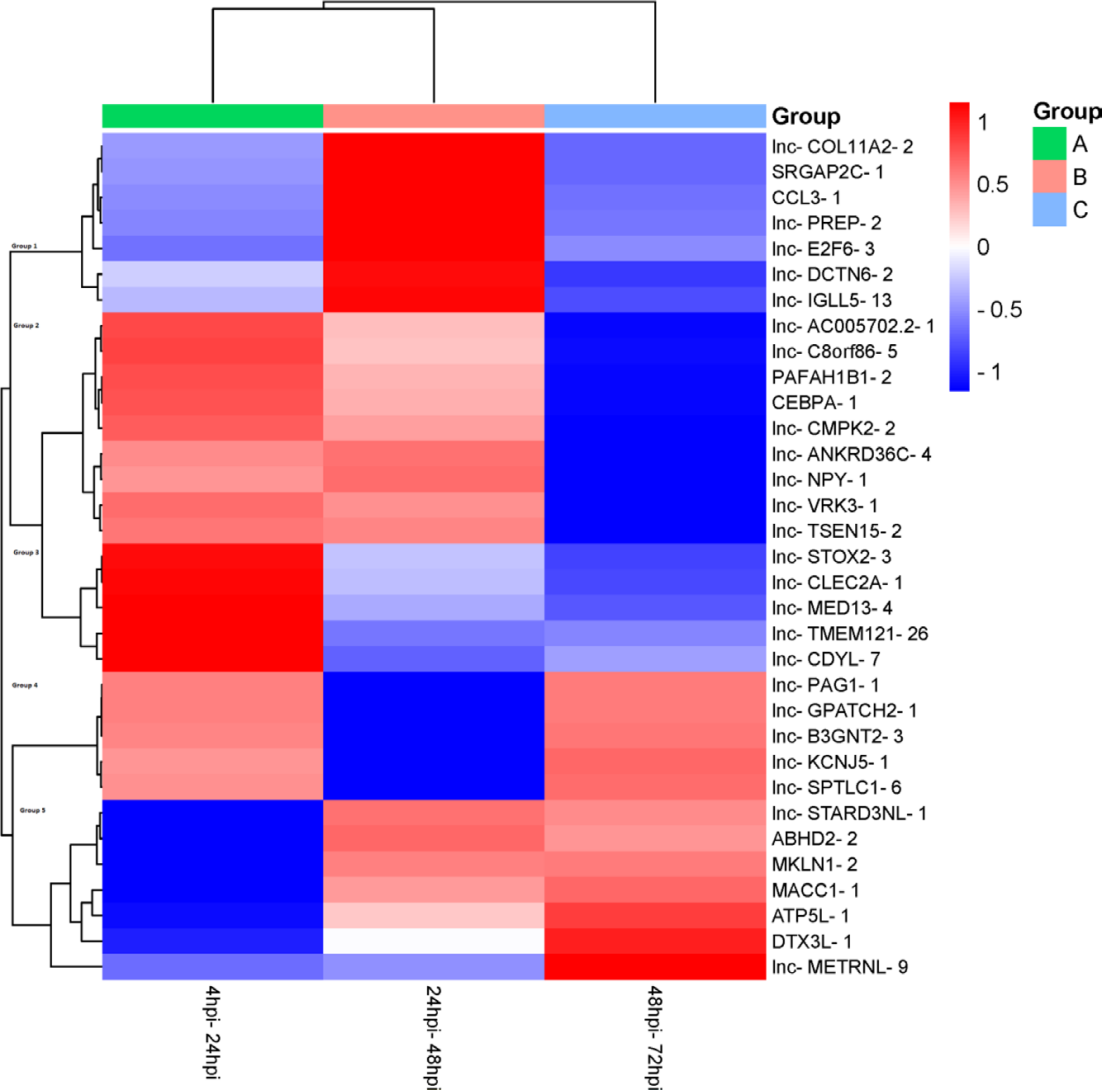
The heatmap displays common differentially expressed lncRNAs across time point comparisons. Columns represent replicates for each comparison, while rows represent individual lncRNAs. The dendrogram above the heatmap indicates hierarchical clustering of the comparisons. Upregulated genes are shown in red, and down-regulated genes are shown in blue. hpi = hours post-infection.

A heat map, as seen in Figure 7, was created to illustrate the expression changes of the 33 common differentially expressed lncRNAs in human macrophages from 4 hpi to 72 hpi. Heatmap analysis identified 5 distinct groups of gene expression patterns. In group 1 antisense lncRNA CCL3-1 showed high expression particularly between 4 hpi and 24 hpi which is associated with the immune system. LncRNAs in this group 2, including lnc-CMPK2-2, displayed a gradual decrease in expression after an initial activation phase at 4 hpi. These genes targets are critical in shaping the immune response. Genes lnc-TMEM121-26 located in group 3, antisense lncRNAs ATP5L-1 and MACC1-1 located in group 5 were found to have immune-related target genes.

### 3.4. Hub gene identification

**Figure 8.**
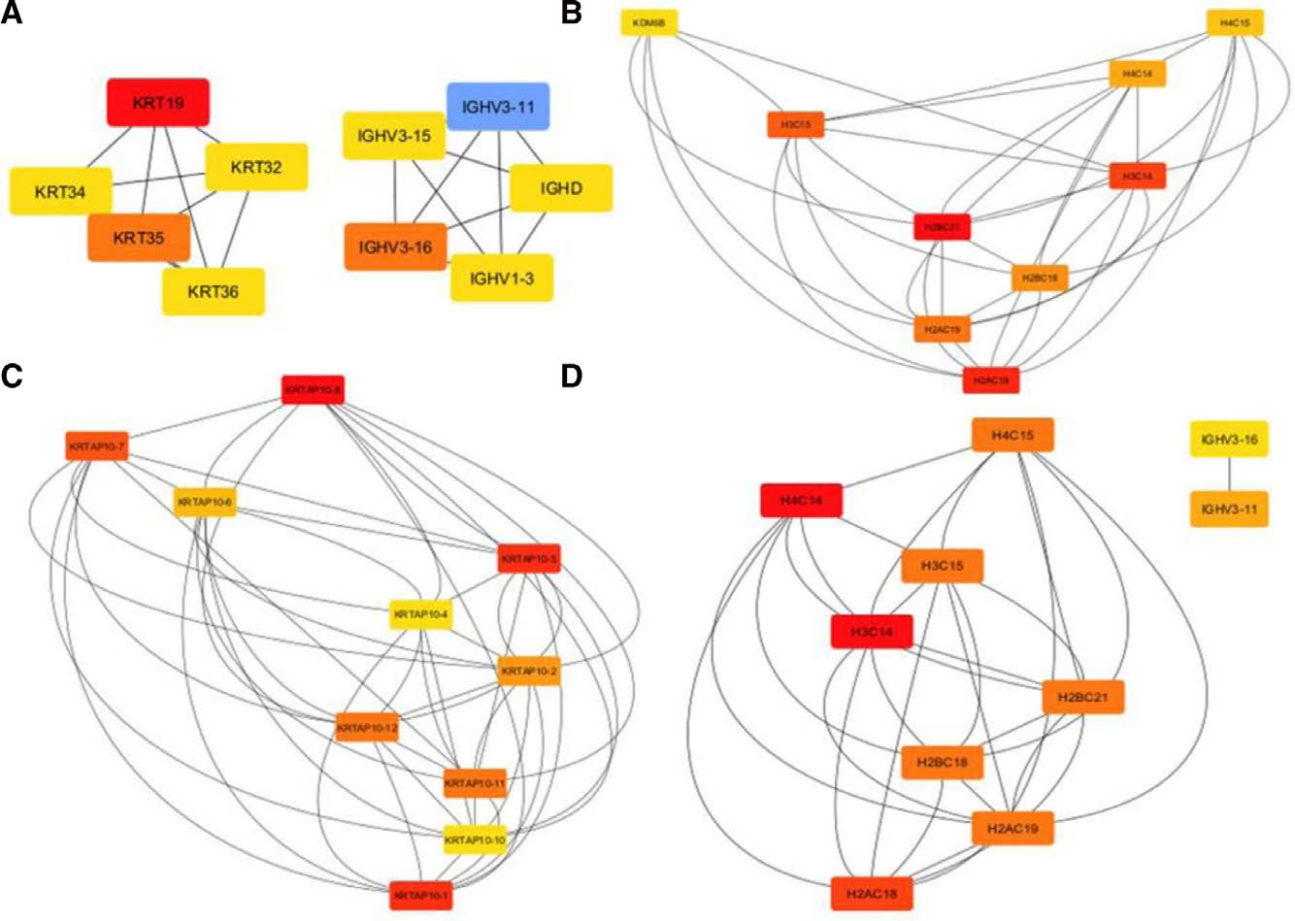
Hub Gene Networks in *Leishmania*-Infected Macrophages at different time points (A) Hub gene network for the 0 to 4-hour interval, showing genes related to cytoskeleton organization and immune response; (B) Hub gene network between 4 and 24 hours, where 8 hub genes are core nucleosome components, and KDM6B is a lysine-specific demethylase involved in transcriptional regulation; (C) Hub gene network between 24 and 48 hours, where all hub genes are associated with keratinization; (D) Hub gene network between 48 and 72 hours, showing genes related to histone modifications and immune response.

Using the STRING tool, cis-target gene-based PPI network was created. Hub genes were identified for each time point using CytoHubba. In all comparisons except 4 hpi vs 24 hpi, 10 hub genes were identified. In all comparisons except 4 hpi vs 24 hpi, 10 hub genes were identified. In the 0 to 4-hours interval, hub genes were associated with cytoskeleton organization and immune response. Between 4 and 24 hours, 8 of the identified hub genes were core components of the nucleosome, with KDM6B identified as a lysine-specific demethylase involved in transcriptional regulation. Interestingly, all hub genes identified between 24 and 48 hours were related to keratinization, while those between 48 and 72 hours were associated with histone modifications and immune response (Fig. 8).

## 4. Discussion

In this study, the temporal expression profiles of DElncRNAs were examined in human macrophages infected with *L major*, and the potential cis-target genes of these DElncRNAs were identified. The RNA-Seq analysis revealed the regulatory profiles of lncRNAs showing differential expression at 4-, 24-, 48-, and 72-hpi, along with their associated target genes. Our findings demonstrate significant variations in lncRNA expression in macrophages during infection, suggesting that these lncRNAs influence various BP and pathways over the course of the infection, playing key roles in regulating the host immune response against the pathogen.

Differentially expressed lncRNAs were identified at 4-, 24-, 48-, and 72-hpi, showing significant changes in the number of DElncRNAs compared to control groups. Additionally, comparisons between different time points revealed a gradual decrease in both upregulated and down-regulated lncRNAs as the infection progressed. This finding suggests that the macrophage response to infection, mediated by lncRNAs, undergoes dynamic changes over time.

Six lncRNAs (*lnc-UNC5D-8*, *lnc-TENM3-1*, *DIRC3-1*, *lnc-MTRNR2L12-10*, *lnc-FAM43A-6*, and *AKAP2-1*) were consistently expressed across all time points, which is particularly noteworthy. All lncRNAs, except *AKAP2-1*, were down-regulated. This suggests that the suppression of certain lncRNAs may play a crucial role in sustaining the macrophage response to *L major* infection. Additionally, common DElncRNAs were identified across the different time points, indicating their potential involvement in regulating macrophage functions at various stages of infection. Understanding the role of these lncRNAs could be important for deciphering the pathophysiology of the infection.

In our study, the cis-target genes located within 300 kb upstream and downstream of the differentially expressed lncRNAs were also identified. This analysis suggests that lncRNAs may control the expression of neighboring protein-coding genes through cis-regulatory mechanisms. A functional enrichment analysis of the cis-target genes was performed to predict the BP and pathways in which these lncRNAs might be epigenetically involved at different time points during the infection.

The rapid activation of the immune response at 4 hpi, compared to the control group, suggests an immediate defense mechanism triggered in response to the pathogen. In the early stages of infection, macrophages recognize pathogens using pattern recognition receptors and bind antigens, a process accelerated by immunoglobulin receptors. The swift engagement of immunoglobulin-mediated responses aims to limit the spread of the parasite and reduce the severity of infection.^[25–27]^ Our data highlight that antigen binding and immunoglobulin-mediated mechanisms were quickly regulated in the first hours of infection, strongly regulated by infected macrophages. The enrichment of processes such as immunoglobulin receptor binding and the prominent involvement of B cell-mediated immunity further underscores the pivotal role of the adaptive immune system during this early phase. These observations align with findings that early immune responses, particularly those involving antibody-mediated pathways, play a critical role in containing parasitic infections.^[28]^ Our findings also suggest that specific lncRNAs identified in this study, such as *lnc-CMPK2-2*, *lnc-TMEM121-26*, and *ATP5L-1*, may have potential therapeutic relevance. Future studies can explore these lncRNAs as biomarkers for monitoring immune responses or as targets for modulating immune mechanisms during *Leishmania* infection. For instance, the role of *lnc-CMPK2-2* in shaping early immune activation highlights its importance in initiating the host defense. Similarly, *ATP5L-1* and *MACC1-1*, with their links to metabolic and immune regulation, provide a foundation for investigating metabolic adaptations of macrophages under parasitic stress. These insights into the functional dynamics of lncRNAs offer promising directions for future therapeutic interventions.

The enrichment analysis also revealed pathways like estrogen signaling and immunoregulatory interactions, suggesting that these pathways might contribute to modulating the host immune response. Interestingly, estrogens, a class of sex hormones, have been linked to immune regulation.^[29]^ The early immune response may also be shaped by transcriptional regulation involving 74 transcription factors and 3 microRNAs (*hsa-miR-9500*, *hsa-miR-6813-5p*, *hsa-miR-6085*), highlighting the complex regulatory networks that control the host’s defense mechanisms at the molecular level.

Between 4- and 24-hpi, the activation of processes related to chemokine receptors and proinflammatory cytokine release indicates an enhanced inflammatory response by infected macrophages aimed at restricting the parasite. This finding aligns with reports in the literature that chemokines and proinflammatory cytokines regulate the infection response, supporting phagocytosis and limiting pathogen spread.^[30,31]^ These molecules not only play roles in recruiting immune cells to the site of infection but also contribute to the activation and maturation of macrophages, reinforcing their capacity to eliminate pathogens. Moreover, during this period, lymphocyte and leukocyte-mediated processes, crucial components of the cellular basis of adaptive immunity, were observed to be active. This suggests that the immune response was transitioning from an innate to a more adaptive phase, where lymphocytes play a critical role in pathogen clearance. The involvement of structures such as the immunoglobulin complex and plasma membrane, identified as key CC, further supports their known roles in pathogen elimination. The significance of immune-related pathways associated with Leishmaniasis during this period highlights the critical transition in immune signaling as the infection progresses. These pathways are likely involved in coordinating the host’s defense mechanisms, facilitating the recognition and targeting of *Leishmania* parasites, and guiding the immune response towards resolution of infection.

Between 24- and 48-hpi, significant changes in immune responses were observed, particularly involving the activation of immunoglobulin-mediated immune responses and miRNA-mediated post-transcriptional gene silencing. This indicates an ongoing regulation of immune responses and modulation of transcriptional processes. Studies have shown that during later stages of *Leishmania* infection in macrophages, gene expression undergoes substantial changes, with proinflammatory responses playing dual roles in controlling the parasite and aiding its survival.^[32–35]^ In the study conducted by Fernandes and colleagues, it was demonstrated that the transcriptome profile of human macrophages infected with *Leishmania* was reprogrammed post-infection, leading to significant changes in cellular responses.^[14]^ Additionally, the identification of CC like the immunoglobulin complex and keratin filaments points to their crucial role in immune response regulation. Immunoglobulin complexes, essential for antigen recognition and immune signaling, are likely involved in enhancing phagocytic activity against the parasite. The presence of keratin filaments, typically linked to structural support, could suggest a role in maintaining cellular integrity during the immune response. The enrichment of pathways such as B cell receptor signaling (KEGG:04662) and *Leishmania*-related phagocytosis emphasizes the involvement of adaptive immune mechanisms in these later stages of infection. B cell receptor signaling plays a critical role in antibody-mediated immunity, facilitating the recognition and clearance of pathogens.

Between 48- and 72-hpi, processes related to the adaptive immune response, immunoglobulin-mediated immune response, and humoral immune response became prominent. This suggests that macrophages may mount a more robust and targeted immune response against the parasite at this stage. While the early phases of infection were dominated by immune activation and inflammatory processes, later stages show the predominance of humoral responses and adaptive immune mechanisms. During this period, CC such as the IgG immunoglobulin complex and extracellular region were active, and MF like immunoglobulin receptor binding and antigen binding were highlighted.

In the 0 to 4-hours interval, the hub genes were primarily associated with cytoskeleton organization and immune response. This suggests that early host responses are geared towards maintaining cellular integrity and potentially to limit the spread of the parasite.

Between 4- and 24-hpi, 8 of the identified hub genes were core components of the nucleosome, indicating a significant shift towards chromatin remodeling and gene regulation. The identification of KDM6B, a lysine-specific demethylase involved in transcriptional regulation, is particularly noteworthy as it points to the modulation of epigenetic marks during infection. In earlier study, they demonstrated that the functional knockdown of KDM6B in an experimental model of visceral leishmaniasis led to a substantial reduction in the parasite burden within infected organs.^[36]^

In the 24 to 48-hours interval, the hub genes were all related to keratinization. This process is closely linked to the function of keratinocytes, which play a pivotal role in the immune response and wound healing in cutaneous leishmaniasis.^[37]^ Furthermore, keratinocyte apoptosis and necrosis have been implicated in the formation of ulcers, a hallmark of cutaneous leishmaniasis pathology. Beyond their involvement in ulceration, keratinocytes also may contribute to the healing of *Leishmania*-related cutaneous wounds by modulating the inflammatory environment at the infection site. Keratinocytes have been shown to either initiate or suppress proinflammatory responses, creating a microenvironment that is uniquely tailored to each *Leishmania* species.^[38]^ The ability to modulate this inflammatory process may significantly influence the course of the disease. Thus, the activation of keratinization-related genes during the 24 to 48-hours interval suggests that keratinocytes play an important role not only in the structural defense of the infected area but also in regulating the immune response and potentially influencing disease progression through species-specific interactions.

Finally, between 48 and 72 hours, hub genes were predominantly associated with histone modifications and immune response. This further highlights the role of epigenetic regulation in shaping the immune response in the later stages of infection. Histone modifications may facilitate gene expression, allowing for a sustained immune response as the infection progresses. These dynamic changes in hub gene activity reflect the complex interplay between cellular processes and immune defenses throughout the course of infection, emphasizing the importance of both structural and regulatory mechanisms in the host’s response to *Leishmania*.

This study has certain limitations. First, the conclusions are based exclusively on computational predictions derived from publicly available RNA-Seq data, with no experimental validation (e.g., qRT-PCR or functional assays) done. Second, the use of a single dataset, despite its excellent quality, may limit its applicability to different *Leishmania* strains or host systems. Finally, the selection of bioinformatics tools and cutoff criteria (such as 300 kb for cis-target genes) may cause bias. Future research that incorporates independent datasets or experimental validation will be crucial to confirm the roles of identified lncRNAs.

## 5. Conclusion

In conclusion, this study provides insights into the time-dependent expression profiles of lncRNAs during *L major* infection and their relationships with potential cis-target genes, enhancing our understanding of how immune responses are regulated at different stages of infection in infected macrophages. The identification of hub genes related to cytoskeleton organization and immune response in the early stages suggests that macrophages mount a rapid and defensive response to the infection. In later stages, processes such as keratinization, histone modifications, and epigenetic regulators play a more significant role. In addition to advancing our understanding of the temporal dynamics of lncRNA expression, this study highlights the potential of these molecules for future applications. The identification of key lncRNAs, such as *lnc-CMPK2-2*, *MACC1-1*, and *ATP5L-1*, opens avenues for exploring their use as biomarkers or therapeutic targets. Future research could focus on validating the functional roles of these lncRNAs in larger cohorts and assessing their potential in developing targeted therapies against *Leishmania* infection.

## Acknowledgments

Thanks to the Çankiri Karatekin University Scientific Research Projects Coordination Unit for supporting this work. Thanks to the anonymous reviewers, academic editors, and editors for their comments and suggestions. We also extend our gratitude to Massive Bioinformatics, a biotechnology and bioinformatics company, for their valuable support and contributions to the data analysis and interpretation in this study.

## Author contributions

**Conceptualization:** Serhat Sirekbasan, Tuğba Gürkök-Tan.

**Data curation:** Tuğba Gürkök-Tan.

**Formal analysis:** Tuğba Gürkök-Tan.

**Funding acquisition:** Serhat Sirekbasan.

**Investigation:** Serhat Sirekbasan, Tuğba Gürkök-Tan.

**Methodology:** Serhat Sirekbasan.

**Project administration:** Tuğba Gürkök-Tan.

**Resources:** Serhat Sirekbasan.

**Software:** Tuğba Gürkök-Tan.

**Supervision:** Serhat Sirekbasan.

**Validation:** Tuğba Gürkök-Tan.

**Visualization:** Serhat Sirekbasan.

**Writing – original draft:** Tuğba Gürkök-Tan.

**Writing – review & editing:** Serhat Sirekbasan, Tuğba Gürkök-Tan.

## Supplementary Material


